# Saponins of Tomato Extract Improve Non-Alcoholic Fatty Liver Disease by Regulating Oxidative Stress and Lipid Homeostasis

**DOI:** 10.3390/antiox12101848

**Published:** 2023-10-12

**Authors:** Ziming Yang, Li Zhang, Jinlei Liu, Albert S. C. Chan, Dianpeng Li

**Affiliations:** 1 Guangxi Key Laboratory of Plant Functional Phytochemicals and Sustainable Utilization, Guangxi Institute of Botany, Guangxi Zhuang Autonomous Region and Chinese Academy of Sciences, Guilin 541006, China; zl@gxib.cn (L.Z.); ljl@gxib.cn (J.L.); ldp@gxib.cn (D.L.); 2 School of Pharmaceutical Sciences, Sun Yat-sen University, Guangzhou 510006, China

**Keywords:** fatty liver, cherry tomatoes, visceral fat, high-fat diet, signaling pathway

## Abstract

The present study investigated the impact of saponins of tomato extract (STE) on non-alcoholic fatty liver disease (NAFLD). The findings demonstrated that introducing STE in NAFLD mice revealed promising results in ameliorating symptoms of oxidative stress, lipid metabolism disorders, visceral fat deposition and fatty liver disease. Moreover, the mechanistic studies have demonstrated that STE delivers its effects by activating adenosine 5′-monophosphate (AMP)-activated protein kinase (AMPK), thereby suppressing downstream protein expression associated with fatty acid synthesis. In such conditions, lipid metabolism can be improved. Simultaneously, STE enhanced nuclear factor erythroid 2-related factor 2 (Nrf2) and entry into the nucleus and initiated the transcription of downstream antioxidant factors, thereby relieving oxidative stress induced by a high-fat diet and lowering oxidative damage to the liver. Such results imply that the administration of STE can be regarded as a viable treatment option for NAFLD, providing a mechanism that can regulate the AMPK and Nrf2 signaling pathways.

## 1. Introduction

Non-alcoholic fatty liver disease (NAFLD) is considered to be the hepatic manifestation of the metabolic syndrome, presenting hepatocellular macrovesicular steatosis as its prominent pathological manifestation [1,2]. Under regular circumstances, fat in the body is decomposed and utilized in the liver but it can be piled up inside the liver when metabolic disturbance or excessive intake occurs, giving rise to fatty liver disease [3]. NAFLD arises from a complex interplay of factors, including metabolic disorders, poor dietary and lifestyle habits, and genetic factors [4]. People with metabolic disease often experience concurrent metabolic disorders that disrupt normal lipid metabolism and trigger excessive fat accumulation in the liver. Additionally, the consumption of excessive calories or excessive carbohydrate intake can also induce overwhelming fat accumulation in the liver. Poor lifestyles, such as a lack of physical activity, a sedentary lifestyle, and sleep, can put the body at risk of NAFLD. Meanwhile, people with family genetic risk factors may be more likely to be afflicted by NAFLD.

The pathogenesis of NAFLD remains unclear and no single fully proven theory can explain all conditions. Oxidative stress is considered one of the most prominent factors in the development of NAFLD and its influencing factors encompass minimized antioxidant enzyme activity and growing free radical content in the body [5]. Oxidative stress is a physiological phenomenon that occurs when the body’s production of deleterious substances, such as free radicals, exceeds the neutralizing capacity of the antioxidant system. These free radicals and other hazardous substances can pose oxidative damage to biological macromolecules such as lipids, proteins, and nucleic acids, inducing the development of diseases or accelerating the aging process [6]. During the development of NAFLD, excessive fat accumulation in the liver can cause oxidative stress [7], inducing the production of free radicals and peroxides which are capable of oxidatively sabotaging cell membranes, making them more permeable and increasing the hepatocytes’ sensitivity to external harmful substances [8]. Mitochondrial function can be compromised by oxidative stress, leading to reduced intracellular adenosine triphosphate (ATP) production and the abnormal accumulation of calcium ions within the mitochondria. These detrimental effects contribute to the exacerbation of hepatocyte damage [8]. Further-reaching investigations have demonstrated that oxidative stress can also boost fatty acid synthesis while minimizing fatty acid oxidation, in turn, facilitating fat accumulation in the liver [9]. Therefore, oxidative stress assumes a negative feedback regulatory function in the process of NAFLD and a reinforced control of oxidative stress can make a contribution to the prevention and treatment of the disease. The nuclear factor erythroid 2-related factor 2 (Nrf2) signaling pathway engages in the oxidative stress response of cells and helps to maintain the intracellular oxidative dynamic balance [10]. By activating the Nrf2 signaling pathway, the expression of multiple antioxidant-related genes is promoted, thereby ameliorating oxidative stress-induced damage and bestowing vital protective mechanisms on the organism. In addition, activation of the Nrf2 signaling pathway can curb fatty acid synthesis in hepatocytes and reduce fat accumulation in hepatocytes by downregulating the expression of pathways related to lipid metabolism [11]. Thus, activation of the Nrf2 signaling pathway earned a seat as a potential option in the treatment of NAFLD. Natural products have been verified to be agonists of the Nrf2 signaling pathway for the treatment of NAFLD. Apigenin has been documented to substantially boost anti-oxidative stress-related factors by activating Nrf2 and reducing hepatic lipid formation in NAFLD mice [12]. Moreover, baicalin was also recorded to harbor the ability to induce the high expression of glutathione-s-transferase (GST), NAD(P)H: quinone oxidoreductase 1 (NQO1), and heme oxygenase 1 (HO1) by upregulating Nrf2 expression, thus achieving its therapeutic effect on NAFLD [13].

In clinical practice, no medication has yet emerged as a completely effective treatment for NAFLD. Consequently, the focus of NAFLD treatment lies within lifestyle modifications, such as reducing carbohydrate and fat consumption, engaging in regular physical activity, and achieving weight reduction [14]. With socioeconomic development and changes in people’s lifestyles and diets, the prevalence of NAFLD is expected to decline exponentially in the upcoming decades and the presence of these patients places an overwhelming burden on both society and families. In such cases, it is imminent to exploit prevailing methods to curb and cure NAFLD. Natural products have captured the attention of the medical community in light of their low toxicity and resistance to drugs. Tomato saponins are a class of water-soluble saponins that are notably abundant in cherry tomatoes [15]. As recorded in literature reports, Esculeoside A and its tomato sapogenol is a better acyl coenzyme A-cholesterol acyltransferase (ACAT) enzyme inhibitor [16]. In our previous analyses, we uncovered that tomato extract is in possession of the capability to preserve lipid metabolism equilibrium [17,18]. In addition, we also unmasked that both water-soluble and lipid-soluble extracts of cherry tomatoes have a potent free radical scavenging effect and that cherry tomato lyophilizing powder is promising in preventing hepatic lipid peroxidation in mice [19]. This study endeavors to shed light on the effects of saponins of tomato extract (STE) on NAFLD mice induced by a high-fat diet and the primary emphasis was given to exploring the potential mechanisms underlying these effects, particularly in terms of reducing oxidative stress and restoring lipid homeostasis, so as to provide a new idea for the prevention and control of NAFLD and establish a theoretical foundation for further research of cherry tomatoes.

## 2. Materials and Methods

### 2.1. Materials

Antibodies against superoxide dismutase 1 (SOD1), glyceraldehyde-3-phosphate dehydrogenase (GAPDH), Nrf2, NQO1, and kelch-like ECH-associated protein-1 (Keap1) were purchased from Cell Signaling Technology (Beverly, MA, USA). The antibody of adenosine 5′-monophosphate (AMP)-activated protein kinase α (AMPKα), p-AMPKα, fatty acid synthetase (FAS), and stearoyl-coenzyme A desaturase-1 (SCD1) were procured from Sigma-Adlrich (Saint Louis, MO, USA). The cherry tomatoes employed in this experiment were harvested from an agroecological garden situated in the Tianyang area, Baise City, Guangxi. Male C57BL/6 mice were obtained from Hunan SJA Laboratory Animal Co., Ltd. (Changsha, China). XT301 high-fat diet and XT304 normal control diet were purchased from Jiangsu Synergetic Pharmaceutical Bioengineering Co., Ltd. (Nangjing, China). The composition and caloric values of the XT301 and XT304 are illustrated in Table 1. Overall, 10% of the energy of the XT304 was from fat and 40% of the energy of the XT301 was from fat.

### 2.2. Preparation of STE

To begin with, 20 kg of fresh cherry tomatoes were thoroughly washed and then crushed into a pulp using a crusher, after which the entire pulp was transferred to an enzymatic vessel. Following this, a pectic enzyme of 0.05% commensurate with the weight of the fresh tomato sample was added and carefully mixed. The enzymatic digestion phase was maintained at a constant temperature of 50 °C for 2 h. The tomato enzymatic solution was first coarsely filtered through 80 mesh filter cloth where the filtrate was derived for separation by centrifugation (2500× *g* for 10 min) in a TGL-16R centrifuge (Heima, Zhuhai, China). Subsequently, the collected supernatant was carefully retrieved while the residue was discarded. The supernatant, obtained in the previous step, was purified using D-101 macroporous resin, the elution was conducted with 80% ethanol, and the resulting eluate was collected and later concentrated, adopting an N-3100 rotary evaporator (Eyela, Tokyo, Japan), and dehydrated adopting a vacuum freeze dryer to remove water. The final product obtained was the STE, with a measured weight of 25.68 g and an extraction rate of approximately 0.13%.

### 2.3. Determination of Total Saponins of Tomato Content

The total saponin content of STE was determined by the spectrophotometric method [20]. A 50 mL measuring flask was utilized to dissolve 5.77 mg of esculeoside A standard in a 50% ethanol solution. Following this, the control solution was meticulously measured in volumes of 0.1, 0.2, 0.3, 0.4, 0.5, and 0.7 mL, with each being transferred into separate stoppered test tubes. After the solvent in the test tube completed evaporation, 0.5 mL of 8% ethanolic vanillin solution and 5.0 mL of 72% sulfuric acid were introduced to shake well and they were heated to 60 °C for 1 h in a water bath and thence cooled in an ice bath for 15 min. In the end, the test tubes were removed and cooled to room temperature and the absorbance of the reaction solution was determined at 530 nm. The linear regression was conducted with the concentration of esculeoside A as the horizontal coordinate and the absorbance optical density (OD) value as the vertical coordinate. The treatment of the STE involved carefully weighing 20 mg of the sample and transferring it to a 100 mL measuring flask. The sample was dissolved in an aqueous solution of 50% ethanol and the above method was also adopted for examining the total saponin content. The total saponin content in STE was calculated according to the standard curve of esculeoside A.

### 2.4. Determination of Esculeoside A Content

The STE and Esculeoside A standard were precisely weighed and then dissolved in methanol using sonication, filtered through a microporous membrane, and reserved for subsequent use. The high-pressure liquid chromatograph-evaporative light scattering detector (HPLC-ELSD) approach, previously adopted for the precise determination of Esculeoside A in tomato samples [21], was applied in this study to confirm the content of Esculeoside A in STE. Agilent 1200 HPLC (Agilent, Palo Alto, CA, USA) chromatographic conditions entailed a ZORBAX SB-C18 column with a column temperature of 30 °C; the mobile phase served as the methanol–water solution in different ratios with gradient elution, the detection time was 25 min in total, the flow rate was set to 0.8 mL/min, and the sample injection volume was designed to be 15 μL. ELSD-2000ES (Alltech, Lexington, KY, USA) was constructed with a drift tube temperature of 100 °C and an airflow rate of 2.7 L/min.

### 2.5. Animal Experimental Design

Approval for all animal procedures in this study was derived from the Research Ethics Committee of the Guangxi Institute of Botany, Guangxi Zhuang Autonomous Region and the Chinese Academy of Science (GXZW2021062002). Following a 7d acclimatization period, mice were randomly divided into two groups in terms of their body weight: the normal diet group (*n* = 24) and the high-fat diet group (*n* = 36). The normal diet group was fed with an XT304 normal control diet and the high-fat diet group was fed with an XT301 high-fat diet for 8 weeks. At the end of the 8-week period, the body weight of all the mice was recorded. For the subsequent experiments, 24 mice from the high-fat diet group were carefully selected based on their body weight. Only those mice whose body mass exceeded the average body mass of mice in the normal diet group by 20% were included. The 24 mice in the normal diet group were randomly split into the normal diet control group (ND) and the normal diet + STE group (ND + STE) in terms of body weight. The 24 mice selected from the high-fat diet group were randomly segmented by the terms of body weight into the high-fat diet control group (HFD) and high-fat diet + STE group (HFD + STE). ND and ND + STE mice were given an XT304 normal control diet and HFD and HFD + STE mice were given an XT301 high-fat diet. In contrast, ND + STE and HFD + STE mice were given STE 200 mg/kg/day by gavage and ND and HFD mice were given an equivalent volume of the vehicle solution by gavage for 8 weeks. All animals were allowed free access to food and water. Weekly weight measurements of the mice were conducted and the administered dose was calibrated according to their body weight.

At the conclusion of the 8-week administration period, the mice were anesthetized with sodium pentobarbital after an 8-h fasting period. Once the mice lost consciousness, they were euthanized by cervical dislocation and their livers, kidneys, perirenal fat, and epididymal fat were obtained. At the same time, the weight of each organ tissue was accurately recorded and the corresponding organ index was calculated using the following formula: liver index = liver weight (mg)/mouse weight (g), kidney index = kidney weight (mg)/mouse weight (g), and visceral fat index = visceral fat weight (mg)/mouse weight (g), where visceral fat mass = epididymal fat mass + perirenal fat mass. The blood samples were loaded into sterilized EP tubes and centrifuged at 2500× *g* for 10 min at 4 °C. Subsequently, the serum was separated and carefully preserved at −20 °C for backup. The right anterior lobe of the liver was homogenized, adopting a glass homogenizer; its homogenate was centrifuged at 3500× *g* for 10 min at 4 °C and then the derived supernatant was stored at −80 °C for backup. One portion of the left lobe of the liver and one portion of the kidney were immersed in 10 times the volume of formalin solution for hematoxylin–eosin (HE) staining. Other than that, another portion of the left lobe of the liver was OCT-embedded and stored at −80 °C for oil red staining. The right posterior lobe of the liver was loaded into sterilized lyophilized tubes and transferred to store in liquid nitrogen for a Western blot assay. The middle lobe of the liver was accurately weighed, loaded into lyophilized tubes, and preserved at −80 °C for measurement of total liver fat, liver triglycerides (TG), and liver total cholesterol (TC) content.

### 2.6. Determination of Biochemical Indicators

The alanine transaminase (ALT), aspartate transaminase (AST), glucose (Glu), creatinine (Cre), blood urea nitrogen (BUN), uric acid (UA), TG, TC, low-density lipoprotein cholesterol (LDL-C), and high-density lipoprotein cholesterol (HDL-C) in the blood were measured according to the corresponding kits (Nanjing Jiancheng, Nanjing, China). SOD activity and malondialdehyde (MDA) content in the liver and blood were determined according to the instructions of the corresponding kits (Nanjing Jiancheng, Nanjing, China).

### 2.7. Oil Red Staining and HE Staining

Oil red staining solution was procured from the Nanjing Jiancheng Institute of Biological Engineering (Nanjing, China). The livers of the mice were oil red stained according to the kit’s methodology [22]. The liver and kidney of the mice were HE stained following previous methods [23].

### 2.8. Determination of Hepatic TG, TC, and Total Fat

The TC and TG in the liver were validated following the instructions of the corresponding kits (Applygen, Beijing, China) [24,25]. Total fat extraction from mouse liver was deployed with the application of the modified Folch method [26]. The liver was homogenized with a dichloromethane–methanol (2:1, *v*/*v*) solution. After homogenization, an oscillating stirrer was employed to stir the homogenized solution for 2 h, allowing the lipids in the tissue to fully dissolve. The resulting precipitate was discarded after the homogenate was subjected to centrifugation at 3000× *g* for 10 min. The liquid phase was retained and mixed with 1/4 of its volume of distilled water through vortexing. Subsequently, the mixture was left to stratify and the solids at the upper layer and interface were aspirated. Then, the liquid phase was mixed with 1/4 of its volume of methanol–water solution (1:1, *v*/*v*) through vortexing and the upper liquid layer along with interfacial material was removed, which was followed by washing repeatedly twice. The organic phase, containing lipids, was then concentrated and dried under reduced pressure utilizing a rotary evaporator. The resulting concentrate was subsequently weighed under an electronic balance.

### 2.9. Extraction of Liver Total Proteins and Nucleoproteins

Extraction of total proteins: frozen liver was retrieved from the liquid nitrogen installation and weighed 50 mg after being chopped. At the subsequent pace, they were added to 200 µL of RIPA lysis buffer and completely crushed using an ultrasonic crusher to extract proteins from the liver. The resulting mixture was then centrifuged at 12,000× *g* for 15 min at 4 °C. The supernatant derived referred to the total proteins. Liver nucleoprotein was extracted under the terms of the instructions of the kit (Invent, Eden Prairie, MN, USA).

### 2.10. Western Blot Analysis

A fraction of the protein supernatant was taken to identify the protein concentration with the implementation of the BCA method and the remaining protein supernatant was subjected to Western blot analysis. Semi-quantitative analysis of the target protein bands was conducted employing an image processing system, with the grayscale values serving as indicators of the protein expression levels. A comparison was carried out between the expression of the protein of interest and the internal reference protein and their relative values were applied to quantify the expression level of the target protein.

### 2.11. Data Statistics

All experimental data were expressed as the mean ± standard deviation. One-way ANOVA was applied to compare the means of multiple samples. Statistical analysis was deployed adopting the GraphPad Prism 8 (GraphPad Software, San Diego, CA, USA) program. Tukey’s procedure was used for post hoc testing, with *p* < 0.05 representing statistically significant differences.

## 3. Results and Discussion

### 3.1. The Content of Total Saponins and Esculeoside A in STE

The data presented in Table 2 indicate that the total saponins of tomato content in STE was 43.12%. In such cases, Esculeoside A constituted 15.63%, making up more than one third of the total saponins of tomato content. These results support the notion that Esculeoside A functions as the primary saponin component in tomatoes, aligning with the findings of the previous study [16,18]. Employing the same method, the content of Esculeoside A in the water-soluble extract of tomato obtained from Qianxi cherry tomatoes in our previous study was revealed to be 17% [18], indicating similarity to the content of Esculeoside A in the extract of this study, demonstrating the stability and reliability of this extraction method.

### 3.2. Effect of STE on Body Weight, Liver Weight, Kidney Weight, and Visceral Fat Weight of Mice

**Figure 1:**
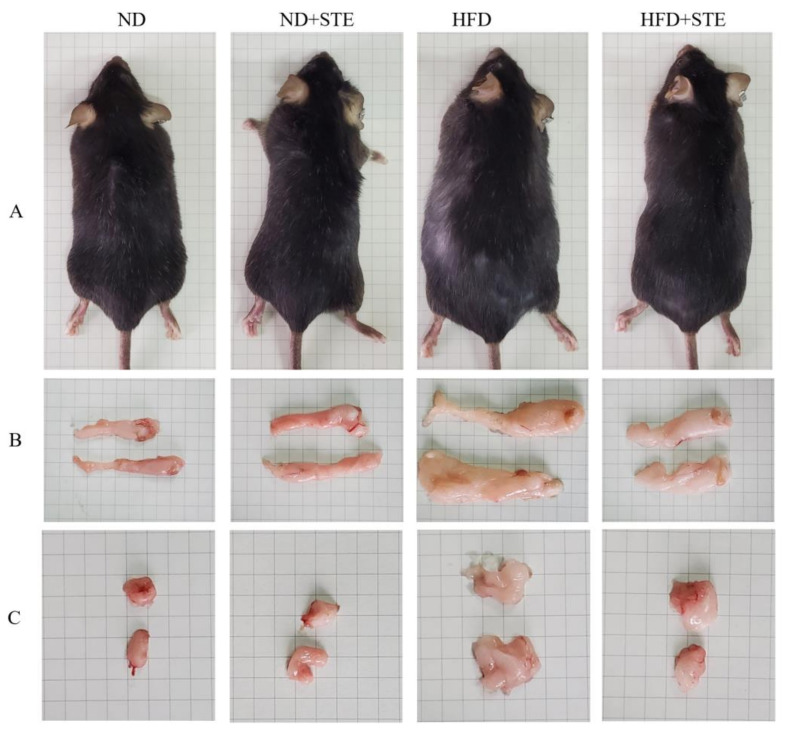
Effect of STE on body weight, perineal fat mass, and epididymal fat mass in high-fat diet-fed mice. (**A**) Mice; (**B**) epididymal fat; (**C**) perineal fat.

The changes in body weight of mice throughout the administration period are elaborated in Table 3 and Figure 1. At the start of the experiment in week 8, both ND and ND + STE mice exhibited an average body weight of approximately 30.3 g. By the end of the experiment at week 16, the average body weight of ND + STE mice reached 33.92 g, representing a 3.58 g gain, while the average body weight of ND mice was at 34.74 g, with a gain of 4.44 g. At the beginning of the experiment in week 8, both HFD and HFD + STE mice displayed an average body weight of approximately 37.6 g which was 20% higher than the mean body weight of ND and ND + STE mice. These results confirmed the successful establishment of an obesity model by the XT301 high-fat diet provided to the mice during the first 8 weeks. By the end of the experiment at week 16, the average body weight of HFD + STE mice was at 39.53 g, disclosing a 2.01 g boost, while the average body weight of HFD mice was at 45.41 g, showing a 7.89 g boost. These conclusions put forward the notion that the weight gain of mice consuming an XT301 high-fat diet was significantly reduced after the administration of STE.

The liver index and kidney index are widely adopted as the indexes to assess the health status of the liver and kidney [27]. Nonetheless, one should bear in mind that the calculation of the liver and kidney index is under the influence of body weight that might potentially trigger bias in the assessment of animals with obese body conditions. In such a case, it is a sheer necessity to take into account various parameters including liver weight, kidney weight, liver index, and kidney index as an attempt to put forth a more accurate assessment. As demonstrated in Table 3, there were no significant differences observed in liver weight, kidney weight, liver index, and kidney index among ND + STE mice and ND mice (*p* > 0.05). The liver weight and liver index of HFD mice were higher than those of ND mice; HFD + STE mice exhibited lower liver weight and liver index values in comparison to HFD mice, suggesting that the supplementation of STE could mitigate hepatomegaly induced by the XT301 high-fat diet. Although the kidney weight of HFD mice did not show differences as compared to ND mice, the kidney index was lower in HFD mice. The kidney weight and kidney index of HFD + STE mice were higher than those of HFD mice. This suggests the need for more far-reaching investigations to shed light on the potential impact of STE on kidney function in the presence of an XT301 high-fat diet.

Visceral fat functions as the adipose tissue distributed around various organs in the abdominal cavity and visceral fat in mice majorly comprises epididymal fat and perirenal fat. Distinguishing from subcutaneous fat, excessive accumulation of visceral fat entails multiple perils, including a mounting risk of committing cardiovascular disease, diabetes mellitus, and cancer [28,29]. As demonstrated in Table 3 and Figure 1, the epididymal fat weight, perirenal fat weight, and visceral fat index of ND + STE mice did not show significant differences in comparison to those of ND mice (*p* > 0.05). HFD mice demonstrated increased epididymal fat weight, perirenal fat weight, and visceral fat index relative to ND mice. In stark contrast, epididymal fat weight, perirenal fat weight, and visceral fat index were relatively declined in HFD + STE mice than in HFD mice. As made clear in Table 3, HFD + STE mice experienced only 12.95% weight loss as compared to HFD mice while the epididymal fat weight, perirenal fat weight, and visceral fat weight were prominently declined by 30.68%, 52.72%, and 37.40%, respectively, highlighting the potential of STE in facilitating reducing visceral fat deposition in mice.

### 3.3. Effect of STE on Liver and Kidney Functions in Mice

**Figure 2:**
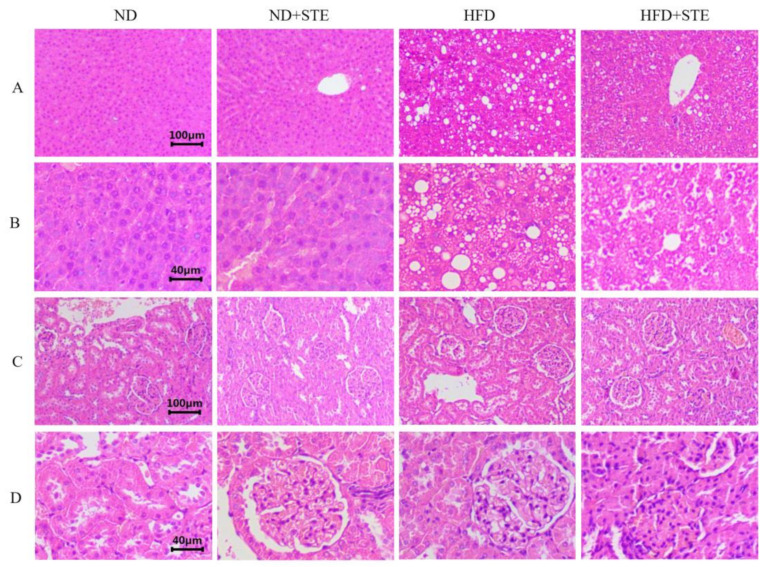
Results of HE staining of liver and kidney. (**A**) Liver damage was assessed using HE staining, bar = 100 µm; (**B**) liver damage was assessed by HE staining, bar = 40 µm; (**C**) kidney damage was assessed using HE staining, bar = 100 µm; (**D**) kidney damage was assessed by HE staining, bar = 40 µm.

ALT and AST are widely employed liver function tests which are released into the bloodstream as hepatocytes are damaged, making them valuable markers for assessing liver function [30]. As demonstrated in Table 4 and Figure 2, the activities of ALT and AST in ND + STE mice did not differ significantly from those in ND mice (*p* > 0.05). The liver HE staining results demonstrated that the liver cells of both ND and ND + STE mice displayed clear structures and intact cell morphology without any evident fat particles in the field of view. The higher ALT and AST levels in HFD mice put forward the fact that the consumption of XT301 high-fat diet in mice might impair liver health. In the liver HE staining results, it was evident that the hepatocytes of HFD mice revealed conspicuous swelling, infiltrated with inflammatory cells, and diffused presence of lipid droplets. Moreover, the observation of round lipid droplet vacuoles in hepatocytes provided compelling evidence that the XT301 high-fat diet could induce liver function damage, resulting in fat accumulation and the occurrence of fatty liver. All of these conditions were alleviated in the livers of HFD + STE mice, demonstrating that STE alleviated the hepatic impairment and hepatic fat accumulation induced by the XT301 high-fat diet.

The assessment of kidney function often involves measuring the levels of Cre and BUN in blood tests, with elevated readings as indications of possible kidney damage [31]. As broken down in Table 4 and Figure 2, there was no significant difference in Cre levels in all experimental groups of mice (*p* > 0.05); BUN levels in HFD mice were lower than those in ND mice. HE staining of the kidney showed that the kidney cells in all experimental groups were structurally intact, normal in size, neatly arranged, and all structures were visible, confirming that the XT301 high-fat diet and the administration of STE did not damage the kidney health. The descent in BUN level was largely attributed to impaired liver function, insufficient protein intake, gastrointestinal bleeding, and abnormal kidney function [32] whereas the drop in BUN level in HFD mice was the result of severe impairment of their liver function. Therefore, the fact that STE could relieve the reduction in BUN caused by XT301 high-fat diet has attested its role in protecting liver function.

### 3.4. Effect of STE on Liver Fat in Mice

**Figure 3:**
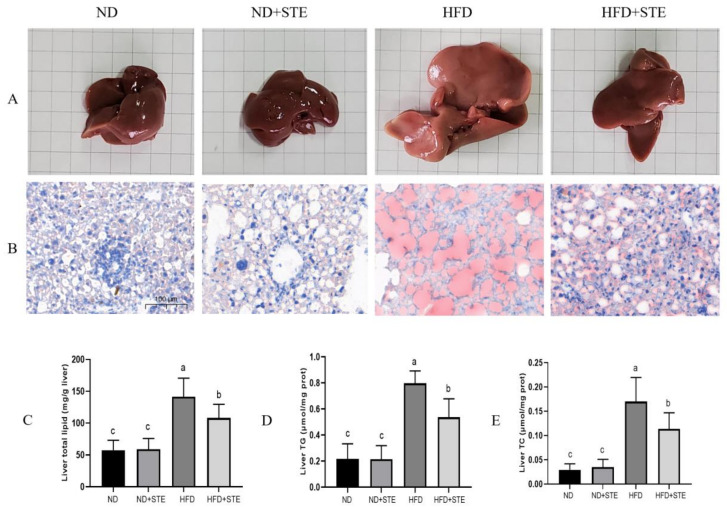
The STE lowered hepatic lipid accumulation in the liver of mice. (**A**) liver; (**B**) liver fat accumulation was assessed by oil red staining, bar = 100 µm; (**C**) liver total lipid content; (**D**) liver TG content; (**E**) liver TC content. Values are expressed as mean ± standard deviation. The same superscript (a, b, or c) in the same column represents no significant differences between values (*p* > 0.05).

After the dissection of the mice in each experimental group, changes in the morphological appearance in the liver were observed using the naked eye (illustrated in Figure 3A): the livers of ND and ND + STE mice presented normal morphology with a reddish-brown color and were elastic, soft in texture, smooth in the peritoneum, and thin at the edges, revealing the absence of any apparent pathological changes; conversely, the liver of HFD mouse was apparently enlarged, yellowish in color, hard in texture, poor in elasticity, and blunt at the edges. In the HFD + STE mouse, notable improvement was observed in the aforementioned symptoms of the liver, characterized by a reduced volume and a color similar to the normal mouse. Nonetheless, the liver exhibited increased hardness, diminished elasticity, and blunt edges. The outcomes of oil red staining of the liver in each experimental group indicated that (Figure 3B) the nuclei of the liver cells of ND and ND + STE mice were stained blue with only a few red lipid droplets; the liver cells of mice on HFD delivered a substantial accumulation of red lipid droplets whereas the liver cells of HFD + STE mice presented a marked reduction in these droplets. Therefore, it is evident that STE delivered promising potential in ameliorating liver fat accumulation. To look deeper, we conducted a comprehensive analysis of the total fat, TG, and TC levels in the liver of mice across all experimental groups. As witnessed in Figure 3C–E, the total fat, TG, and TC contents in the liver of ND + STE mice were not significantly different from those of ND mice (*p* > 0.05). The total fat, TG, and TC contents in the liver of the HFD mice were higher than those of the ND mice. Meanwhile, the decrease in the total fat, TG, and TC contents in the liver of HFD + STE mice, in comparison to HFD mice, once again validates the positive effect of STE in mitigating liver fat accumulation caused by XT301 high-fat diet, highlighting its therapeutic implications for NAFLD.

### 3.5. Effects of STE on Blood Glucose, UA, and Lipids in Mice

**Figure 4:**
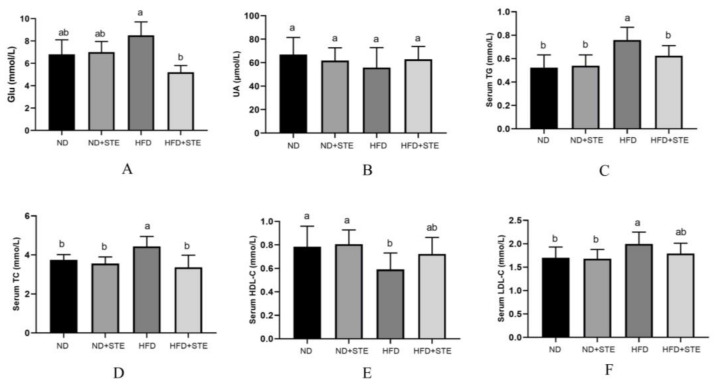
Glu, UA, TG, TC, HDL-C, and LDL-C levels in the serum of mice: (**A**) Glu content in the serum of mice; (**B**) UA content in the serum of mice; (**C**) TG content in the serum of mice; (**D**) TC content in the serum of mice; (**E**) HDL-C content in the serum of mice; (**F**) LDL-C content in the serum of mice. Values are expressed as mean ± standard deviation. The same superscript (a, or b) in the same column represents no significant differences between values (*p* > 0.05).

Past investigations have expounded that prolonged consumption of a high-fat diet serves as a culprit of triggering metabolic disorders and insulin resistance, which in turn heightens the risk of diabetes mellitus [33]. Furthermore, consumption of a high-fat diet has been associated with reduced excretion of UA which can escalate to increased levels of serum UA [34]. As observed in Figure 4A, the levels of glucose were boosted in HFD mice as compared to ND mice while they were reduced in HFD + STE mice as compared to HFD mice, suggesting that prolonged consumption of XT301 high-fat diet can contribute to elevated blood glucose levels whereas STE possesses a hypoglycemic effect. The hypoglycemic function of STE may be relevant to its high content of esculeoside A. Our previous study illustrated that esculeoside A comes into effect by lowering fasting plasma glucose and improving glucose tolerance in db/db diabetic mice [27]. As explicitly demonstrated in Figure 4B, there were no significant differences in UA levels in all experimental groups (*p* > 0.05), indicating that the XT301 high-fat diet does not provoke UA metabolic disorders in mice and that STE has no effect on UA metabolism.

Long-term intake of a high-fat diet not only contributes to fat accumulation in the liver but also disrupts the balance of blood lipid metabolism [35]. From Figure 4C–F, it is illustrated that the levels of TG, TC, HDL-C, and LDL-C in the blood of ND + STE mice were not significantly different from those of ND mice (*p* > 0.05). The HDL-C level was dropped and the TC, TG, and LDL-C levels were promoted in HFD mice by comparison to ND and HFD + STE mice, revealing that the XT301 high-fat diet can bring about blood lipid metabolism disorders in mice and STE can maintain lipid homeostasis.

### 3.6. Effects of STE on SOD Activity and MDA Content in Livers and Blood of Mice

**Figure 5:**
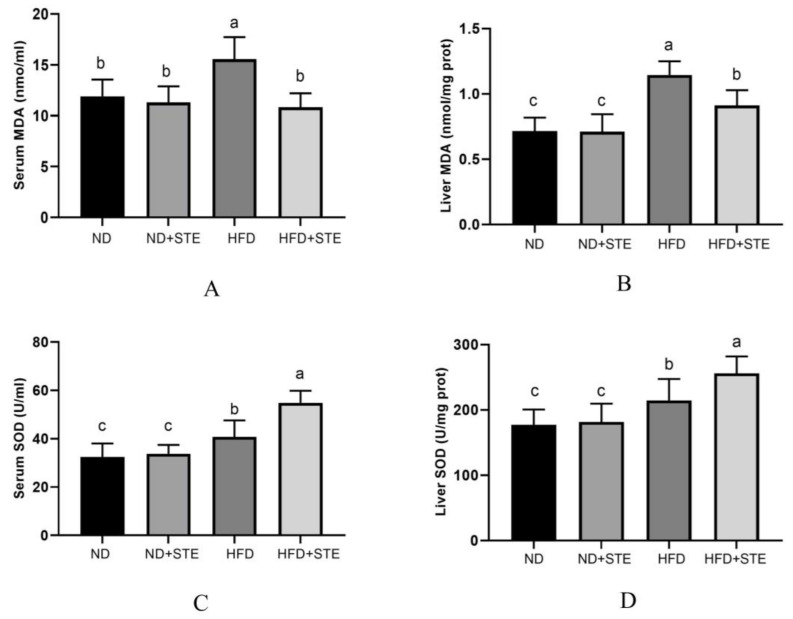
SOD and MDA levels in the serum and liver of mice. (**A**) MDA content in the serum of mice; (**B**) MDA content in the liver of mice; (**C**) SOD activity in the serum of mice; (**D**) SOD activity in the liver of mice. Values are expressed as mean ± standard deviation. The same superscript (a, b, or c) in the same column represents no significant differences between values (*p* > 0.05).

Oxidative stress is identified as one of the vital factors in the development of NAFLD and our previous study uncovered that cherry tomato lyophilized powder poses the influence of enhancing antioxidant activity and preventing lipid peroxidation in the liver [19]. The main character of SOD is to convert O_2_^−^ into O_2_ and H_2_O_2_, stemming their accumulation in cells and preventing oxidative damage [36]. MDA is frequently used as an indicator to gauge the level of oxidative stress [37]. Increased-scale concentrations of MDA can result in oxidative damage to cell membranes, proteins, nucleic acids, and other biomolecules by binding with them, with the level of MDA in the liver reflecting the degree of lipid peroxidation in hepatocytes. As unmasked in Figure 5A,B, the liver and blood of HFD mice exhibited more MDA contents in comparison to ND and HFD + STE mice. Notably, the administration of STE ameliorated the oxidative stress. In response to oxidative stress, the body augments its antioxidant capacity by upregulating the activity of antioxidant enzymes (e.g., SOD) to protect the body from oxidative damage [38], which was confirmed by our experimental results. As revealed in Figure 5C,D, SOD activity in the liver and blood of HFD mice was significantly higher in contrast to ND mice (*p* < 0.05) but reduced in contrast to HFD + STE mice, making it clear that mice can improve the activity of antioxidant enzymes in the body to resist oxidative damage from the high-fat diet but the protective mechanisms may fall short in their ability to adequately counter the external damage inflicted; the STE can further reduce the oxidative stress by upregulating the antioxidant enzyme activity.

### 3.7. Effect of STE on the Nrf2 Pathway

**Figure 6:**
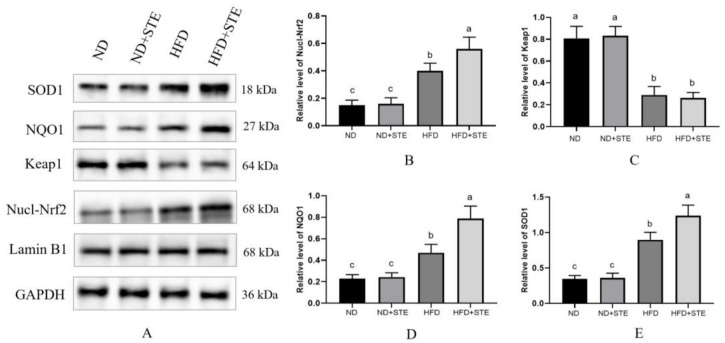
Effects of STE on the protein expression of the Nrf2 signaling pathway in the liver of mice. (**A**) The protein expression of Nucl-Nrf2, Keap1, NQO1, and SOD1; (**B**) quantification of protein levels of Nucl-Nrf2; (**C**) quantification of protein levels of Keap1; (**D**) quantification of protein levels of NQO1; (**E**) quantification of protein levels of SOD1. Values are expressed as mean ± standard deviation. The same superscript (a, b, or c) in the same column represents no significant differences between values (*p* > 0.05).

Activation of the Nrf2 signaling pathway serves as a vital protective mechanism against oxidative stress. Under conditions of oxidative homeostasis, Nrf2 is predominantly localized in the cytoplasm and is regulated by the Keap1 which also inhibits its transmembrane transport [39,40]. Upon exposure to oxidative stress, Keap1 loses or weakens its ability to ubiquitinate Nrf2, leading to the stabilization and accumulation of Nrf2; at the same time, Keap1 undergoes conformational alterations, bringing about the dissociation of Nrf2 from Keap1 and Nrf2 translocates from the cytoplasm to the nucleus where it interacts with the antioxidant response element (ARE), thus initiating transcription of downstream antioxidant factors such as SOD, HO-1, and NQO1 [10,41]. As revealed in Figure 6A,B, HFD mice revealed elevated protein expression of Nrf2 in the nucleus compared to ND mice, demonstrating that oxidative stress generated by an XT301 high-fat diet can trigger Nrf2 activation; Nrf2 protein expression in the nucleus was further elevated in HFD + STE mice when compared to HFD mice, uncovering that STE functions by helping Nrf2 to enter the nucleus. Nevertheless, there was no distinguishment in the protein expression of Nrf2 in the nucleus between ND + STE mice and ND mice, indicating that STE may have the function of helping Nrf2 to enter the nucleus only under oxidative stress. As witnessed in Figure 6A,C, the expression of Keap1 diminished in HFD mice in contrast to ND mice. Consequently, this descending Keap1 expression reduced the degradation of Nrf2 and the inhibition of Nrf2 nuclear translocation. There were also no differences in Keap1 expression when comparing HFD + STE with HFD mice, proposing that the role of STE in helping Nrf2 to enter the nucleus may not be correlated to Keap1. NQO1 exerts its essential function in cells by mediating the transfer of electrons between quinone and NAD(P)H. This enzymatic activity is a crucial defense mechanism that protects cells against the damaging effects of oxidative stress [42]; another crucial antioxidant enzyme is SOD. As shown in Figure 6A,D,E, the protein expressions of NQO1 and SOD1 were markedly mounted in HFD mice compared with ND mice but were lower than that of HFD + STE mice, uncovering that the XT301 high-fat diet activates Nrf2, which in turn initiates the expressions of downstream antioxidant factors; the STE further increased the expressions of antioxidant factors, enhancing the antioxidant activity of the body and reducing the damage caused by a high-fat diet.

### 3.8. Effect of STE on AMPK Pathway

**Figure 7:**
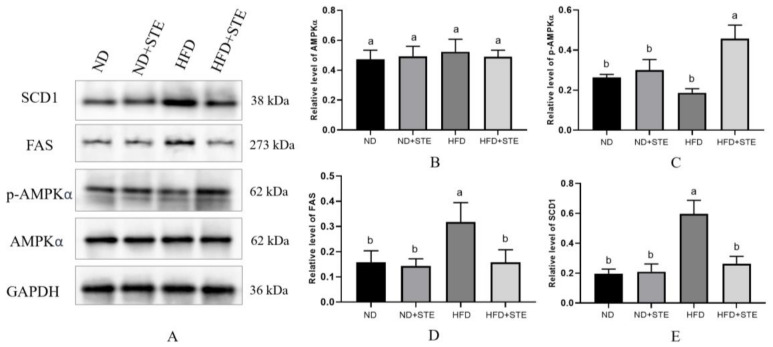
Effects of STE on the protein expression of the AMPK signaling pathway in the liver of mice. (**A**) The protein expression of AMPKα, p-AMPKα, FAS, and SCD1; (**B**) quantification of protein levels of AMPKα; (**C**) quantification of protein levels of p-AMPKα; (**D**) quantification of protein levels of FAS; (**E**) quantification of protein levels of SCD1. Values are expressed as mean ± standard deviation. The same superscript (a, or b) in the same column represents no significant differences between values (*p* > 0.05).

AMPK is now widely recognized as the crucial protein responsible for balancing energy supply with demand [43]. Activated AMPK regulates a variety of metabolic processes. These include the suppression of fatty acid and triglyceride synthesis, alongside the promotion of triglyceride hydrolysis [44]. Esculeoside A, which constitutes a major portion of STE, has been identified as an AMPK agonist in previous investigations [27]. It is therefore reasonable to speculate that STE may activate AMPK. From Figure 7A,C, p-AMPKα protein expression was diminished in HFD mice in comparison with ND mice, proposing that prolonged consumption of an XT301 high-fat diet in mice can curb AMPK and may bring about the imbalance of energy metabolism, resulting in abnormal lipid metabolism and weight gain in mice. However, the p-AMPKα protein expression was boosted in HFD + STE mice as compared with HFD mice, demonstrating that STE has an activating effect on AMPK. Activation of AMPK was reported to cause nuclear accumulation of Nrf2 [45]. This may offer an explanation for the effect of STE in promoting Nrf2 to enter the nucleus. FAS is a pivotal rate-limiting enzyme in the synthesis of fatty acids that catalyzes the synthesis of long-chain fatty acids from malonyl-CoA and acetyl-CoA [46]. SCD1 serves as a core rate-limiting enzyme for the synthesis of monounsaturated fatty acids in hepatocytes [47]. FAS and SCD1 act as downstream transcription factors of AMPK; activation of AMPK can inhibit FAS and SCD1 expressions. In accordance with Figure 7A,D,E, an XT301 high-fat diet elevated the protein expressions of hepatic FAS and SCD1 in mice and STE decreased the protein expressions of FAS and SCD1, which is an obvious testament that STE treatment of mice activated AMPK, thereby hindered the downstream FAS and SCD1 protein expression associated to fatty acid synthesis and thus lowering the production of fatty acids to improve lipid metabolism.

## 4. Conclusions

In this study, STE was extracted from cherry tomatoes and esculeoside A was attested to be its major saponin component. The model of NAFLD, established by administering mice the XT301 high-fat diet, exhibited oxidative stress, lipid metabolism disorders, visceral fat deposition, and fatty liver, which can be alleviated by the application of STE. The mechanism underlying the improvement in NAFLD by STE was elucidated in a follow-up study which highlighted its regulation of the AMPK and Nrf2-Keap1 signaling pathways. STE activates AMPK, thereby suppressing downstream FAS and SCD1 protein expression which is correlated to fatty acid synthesis, hence upgrading lipid metabolism. STE functions to enhance Nrf2 entry into the nucleus and initiate transcription of downstream antioxidant factors SOD and NQO1, thereby alleviating XT301 high-fat diet-induced oxidative stress and reducing oxidative damage of the liver. The study’s findings provide an experimental underpinning for the regulation of oxidative stress and lipid metabolism by STE, setting a scrutiny-withstanding foundation for its use as a functional food for the treatment of NAFLD, thereby enhancing the development value of cherry tomatoes.

## Figures and Tables

**Table 1:** Composition of experimental diets.

Item	XT304 Normal Control Diet	XT301 High-Fat Diet
Ingredient	amounts (g)	amounts (g)
Casein	200.00	200.00
L-Cystine	3.00	3.00
Corn starch	504.00	-
Fructose	-	200.00
Maltodextrin	100.00	100.00
Sucrose	100.00	100.00
Cellulose	50.00	50.00
Soybean oil	25.00	25.00
Lard	20.00	20.00
Primex Shortening	-	135.00
Mineral mix S10026B	50.00	50.00
Vitamin mix V10001C	1.00	1.00
Choline bitartrate	2.00	2.00
Cholesterol	-	18.00
Formula	proportion (%)	proportion (%)
Protein	19.24	22.45
Carbohydrate	66.82	44.36
Fat	4.27	19.91
Energy	proportion (%)	proportion (%)
Protein	20.11	20.12
Carbohydrate	69.85	39.74
Fat	10.04	40.14
	kal/g	kal/g
Diet	3.83	4.46

**Table 2:** Bioactive components of STE.

Item	STE
Total saponins of tomato (g/100 g)	43.12 ± 1.31
Esculeoside A (g/100 g)	15.63 ± 0.42

Values were the mean of triplicate experiments ± standard deviation.

**Table 3:** STE effect on body weight, organ weight, and visceral fat weight in mice.

Item	ND	ND + STE	HFD	HFD + STE
Initial weight (g)	30.32 ± 0.89 ^b^	30.33 ± 0.91 ^b^	37.63 ± 0.92 ^a^	37.61 ± 1.05 ^a^
Final weight (g)	34.74 ± 1.13 ^c^	33.92 ± 0.92 ^c^	45.41 ± 3.21 ^a^	39.53 ± 2.62 ^b^
Weight gain (g)	4.44 ± 0.72 ^b^	3.58 ± 0.83 ^b^	7.89 ± 0.81 ^a^	2.01 ± 0.33 ^c^
Liver (g)	1.30 ± 0.09 ^c^	1.29 ± 0.10 ^c^	2.01 ± 0.26 ^a^	1.66 ± 0.20 ^b^
Liver index (mg/g)	36.87 ± 1.59 ^b^	37.24 ± 1.48 ^b^	44.51 ± 3.56 ^a^	41.25 ± 3.18 ^a^
Kidney (g)	0.40 ± 0.02 ^a^	0.42 ± 0.03 ^a^	0.40 ± 0.06 ^a^	0.45 ± 0.07 ^a^
Kidney index (mg/g)	11.28 ± 0.38 ^a^	11.45 ± 0.51 ^a^	8.80 ± 1.05 ^b^	11.34 ± 0.02 ^a^
Perirenal fat pad (g)	0.23 ± 0.06 ^c^	0.24 ± 0.06 ^c^	1.10 ± 0.21 ^a^	0.52 ± 0.15 ^b^
Epididymal fat pad (g)	0.90 ± 0.20 ^c^	0.92 ± 0.23 ^c^	2.51 ± 0.57 ^a^	1.74 ± 0.47 ^b^
Visceral fat (g)	1.13 ± 0.26 ^c^	1.19 ± 0.33 ^c^	3.61 ± 0.43 ^a^	2.26 ± 0.57 ^b^
Visceral fat index (mg/g)	32.17 ± 6.82 ^c^	34.15 ± 4.41 ^c^	80.23 ± 7.64 ^a^	56.60 ± 10.69 ^b^

Values were expressed as mean ± standard deviation. Liver index = liver weight (mg)/mice weight (g), kidney index = kidney weight (mg)/mice weight (g), visceral fat index = visceral fat weight (mg)/mice weight (g). The same superscript (a, b, or c) in the same line represents no significant differences between values (*p* > 0.05).

**Table 4:** The effect of STE on ALT, AST, BUN, and Cre in mice.

Item	ND	ND + STE	HFD	HFD + STE
ALT (IU/L)	5.41 ± 2.14 ^c^	5.22 ± 2.32 ^c^	19.87 ± 4.88 ^a^	11.68 ± 3.52 ^b^
AST (IU/L)	4.12 ± 1.53 ^c^	4.56 ± 1.38 ^c^	15.01 ± 3.25 ^a^	9.83 ± 2.12 ^b^
BUN (mmol/L)	11.36 ± 2.18 ^a^	11.13 ± 1.98 ^a^	8.74 ± 0.88 ^b^	9.89 ± 0.95 ^ab^
Cre (μmol/L)	4.34 ± 0.51 ^a^	4.21 ± 0.38 ^a^	4.75 ± 0.86 ^a^	4.66 ± 0.45 ^a^

Values were expressed as mean ± standard deviation. The same superscript (a, b, or c) in the same line represents no significant differences between values (*p* > 0.05).

## Data Availability

Data is contained within the article.
